# Supplementary material for: Zur and zinc increase expression of E. coli ribosomal protein L31 through RNA-mediated repression of the repressor L31p
Source: Nucleic Acids Res. 2022 Dec 19;50(22):12739–53. doi: 10.1093/nar/gkac1086 (PMC9825181; doi:10.1093/nar/gkac1086)
Supplement: gkac1086_Supplemental_Files [file gkac1086_supplemental_files.zip › Rasmussen_L31_Zur_zinc_repression_SI-revision-SUBMIT.pdf]

Supplementary Information For

Zur and Zinc Increase Expression of *E. coli* Ribosomal Protein L31 Through RNA-Mediated Repression of the Repressor L31p

Rebecca A. Rasmussen[1,2], Suning Wang[2,3], Jeannie M. Camarillo[4], Victoria Sosnowski[4], Byoung-Kyu Cho[4,5], Young Ah Goo[4,5], Julius B. Lucks[1,2,6,7]*, Thomas V. O'Halloran[2,3,8,9]*

[1]Interdisciplinary Biological Sciences Graduate Program, Northwestern University, Evanston, IL 60208, USA
[2]Chemistry of Life Process Institute, Northwestern University, Evanston, Illinois, 60208, United States of America.
[3]Department of Chemistry, Northwestern University, Evanston, Illinois, 60208, United States of America.
[4]Northwestern Proteomics Core, Northwestern University, Evanston, Illinois, 60208, United States of America.
[5]Mass Spectrometry Technology Access Center, Washington University in St Louis, School of Medicine
[6]Department of Chemical and Biological Engineering, Northwestern University, Evanston, IL 60208, USA
[7]Center for Synthetic Biology, Northwestern University, Evanston, IL 60208, USA
[8]Department of Chemistry, Michigan State University, East Lansing, MI 48824, USA.
[9]Department of Microbiology & Molecular Genetics, Michigan State University, East Lansing, MI 48824, USA.
*Corresponding authors

Table of Contents

Figure	Page
**Figure S1. *In vivo* GFP assay growth calibration in WT and *Δzur E. coli* cells grown in LB.**	2
**Figure S2. PAGE gels of monolith chromatography fractions of purified ribosomes from A.) WT and B.) *Δzur E. coli* cells.**	3
**Figure S3. *In vivo* reporter gene assays for fluorescence and growth of strains with *l31p* or *l36p* knocked out.**	4
**Figure S4 Growth of strains in zinc-deficient and zinc sufficient conditions.**	6
**Figure S5. Predicted secondary structures of the *l31* 5'UTR and its mutants.**	7-8
**Figure S6. The top bulge of the *l31* 5'UTR stem loop is not important for the regulation of L31-sfGFP by *zur*.**	9
**Figure S7. *In vivo* sfGFP gene assays including plasmids that constitutively overexpress ribosomal proteins.**	11
**Table S1. Plasmids used in this study.**	12
**Table S2. Primers used in RT-qPCR.**	13
**Table S3. Summary of *in vivo* assay figures.**	13
**Table S4. ICP-MS measurements of Zn in LB media used in zinc-depletion experiments.**	13
**Table S5. Quantitative cycle threshold values (Cq) from RT-qPCR measurements on WT and *Δzur* cells with sfGFP-plasmids.**	13-15

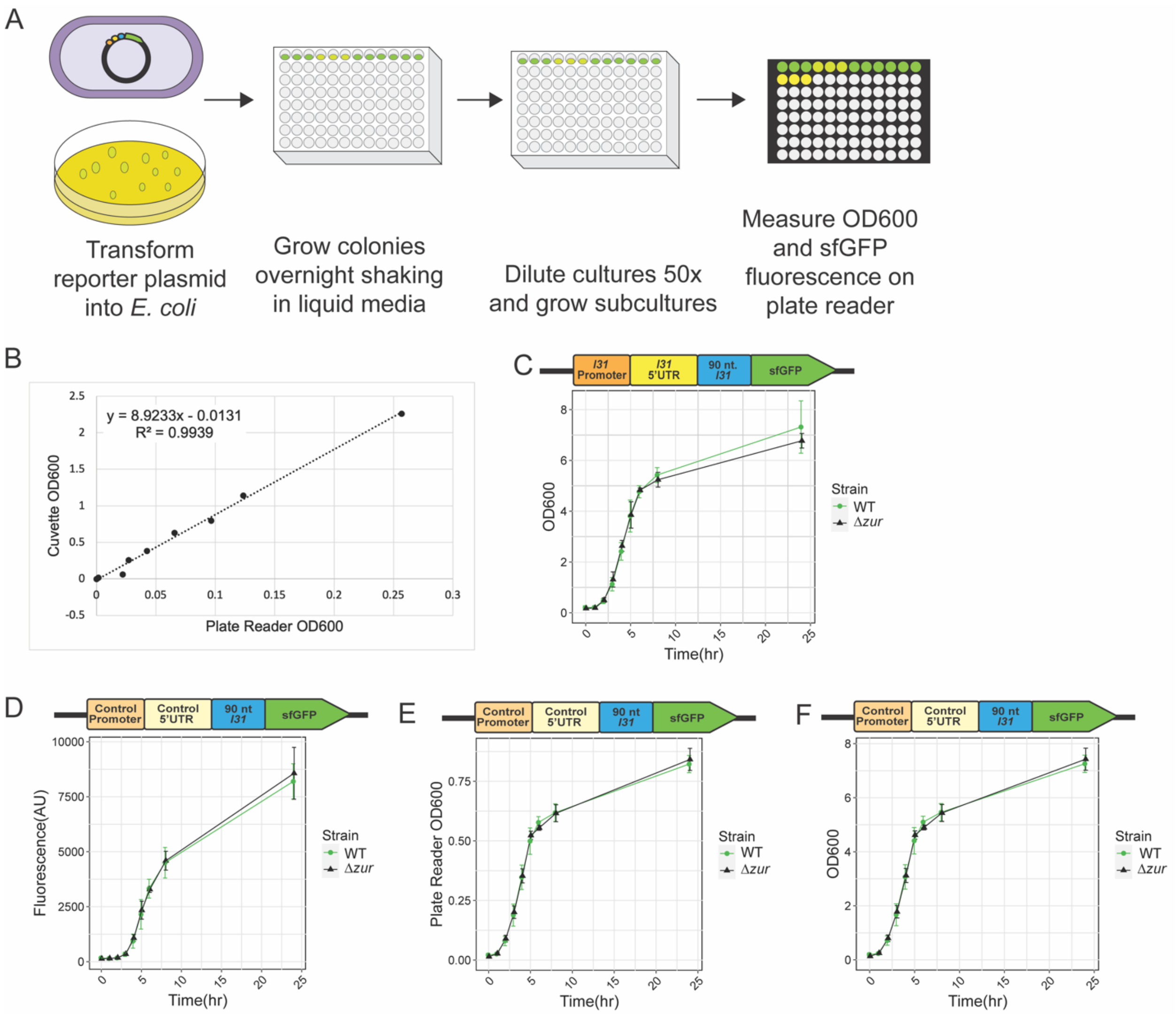

Figure S1. *In vivo* sfGFP assay growth calibration in WT and *Δzur E. coli* cells grown in LB. A.) Schematic for experiment setup of live cell reporter gene assays. B.) Calibration curve generated by diluting a saturated WT *E. coli* culture in LB and measuring both on the plate reader and a cuvette in a spectrophotometer. The linear regression calculation was generated by Microsoft Excel. C.) OD_{600} of cells with L31-sfGFP plasmid over time, using the equation from panel B to convert the plate reader measurements into the more standard spectrophotometer values. D.) Fluorescence from control-sfGFP plasmid in cells from 0-24 hours, measured on a plate reader. E.) OD_{600} of cells with L31-sfGFP plasmid over time measured on the plate reader, and F.) converted to standard growth values using the equation from panel B. The bars indicate averages of three biological replicates (independent experiments), each performed with three technical replicates (cultures per experiment) for a total of nine data points (n=9.) The error bars represent standard deviation of the mean.

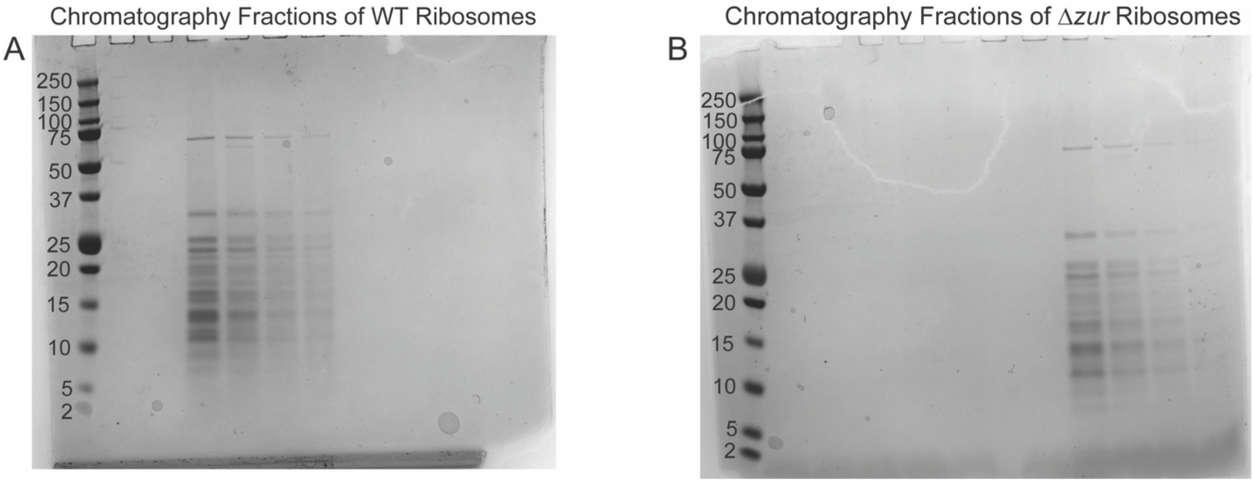

Figure S2. PAGE gels of monolith chromatography fractions of purified ribosomes from A.) WT and B.) Δ*zur E. coli* cells.

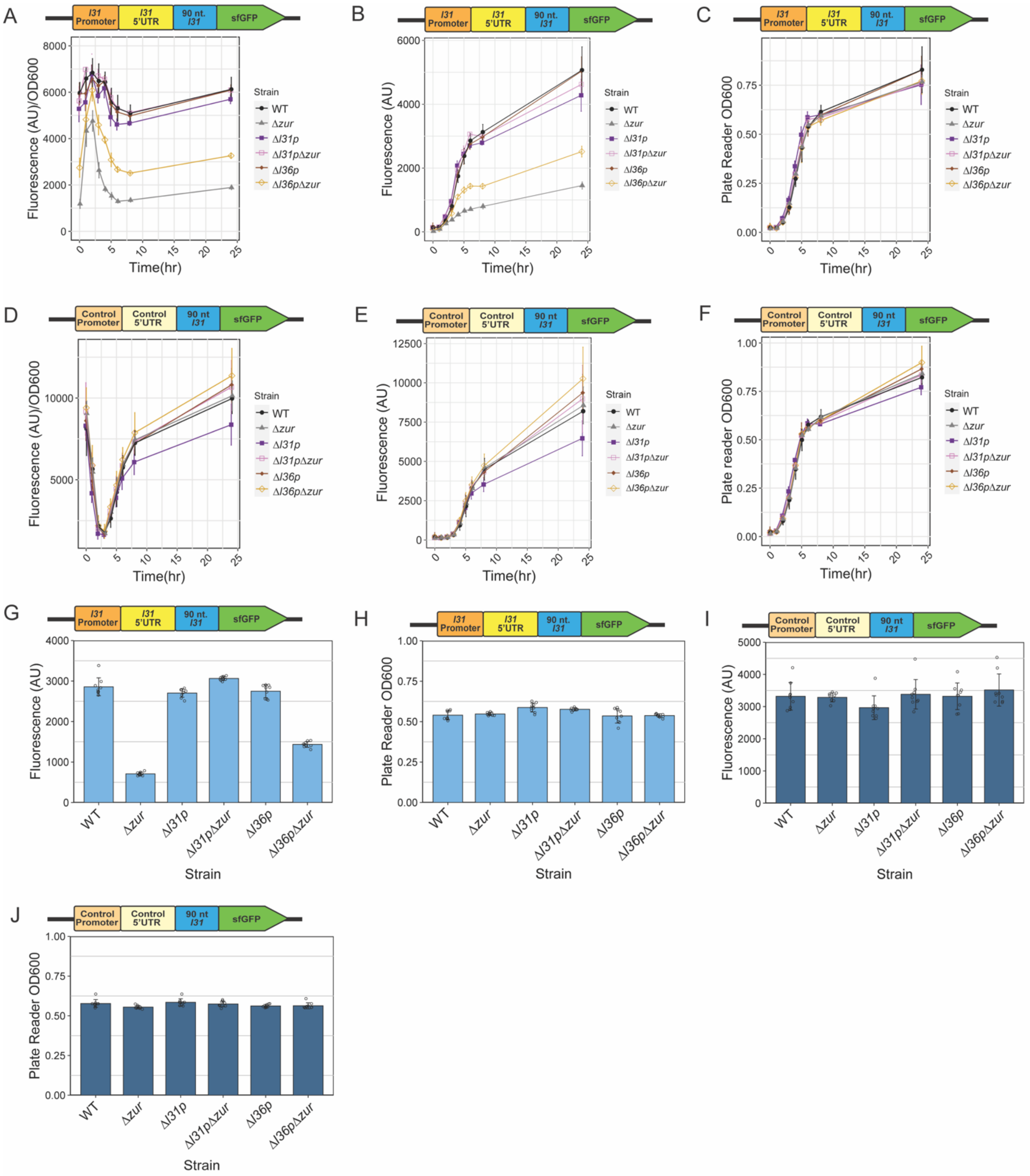

Figure S3. *In vivo* reporter gene assays for fluorescence and growth of strains with *I31p* or *I36p* knocked out. A.) Fluorescence/OD_{600}, B.) Fluorescence (AU), and C.) Plate reader OD_{600} time-course of *E. coli* knockout strains with L31-sfGFP plasmid grown in LB media. D.) Fluorescence/OD_{600}, E.) Fluorescence (AU), and F.) Plate reader OD_{600} time-course of *E. coli* knockout strains with control-sfGFP plasmid grown in LB media. G.) Plate reader fluorescence and H.) OD_{600} of strains grown in LB with L31-sfGFP plasmid at the 6 hr timepoint. I.) Plate reader fluorescence and J.) OD_{600} of strains grown in LB with control-sfGFP plasmid at the 6 hr timepoint. The bars indicate

averages of three biological replicates (independent experiments), each performed with three technical replicates (cultures per experiment) for a total of nine data points (n=9.) The error bars represent standard deviation of the mean.

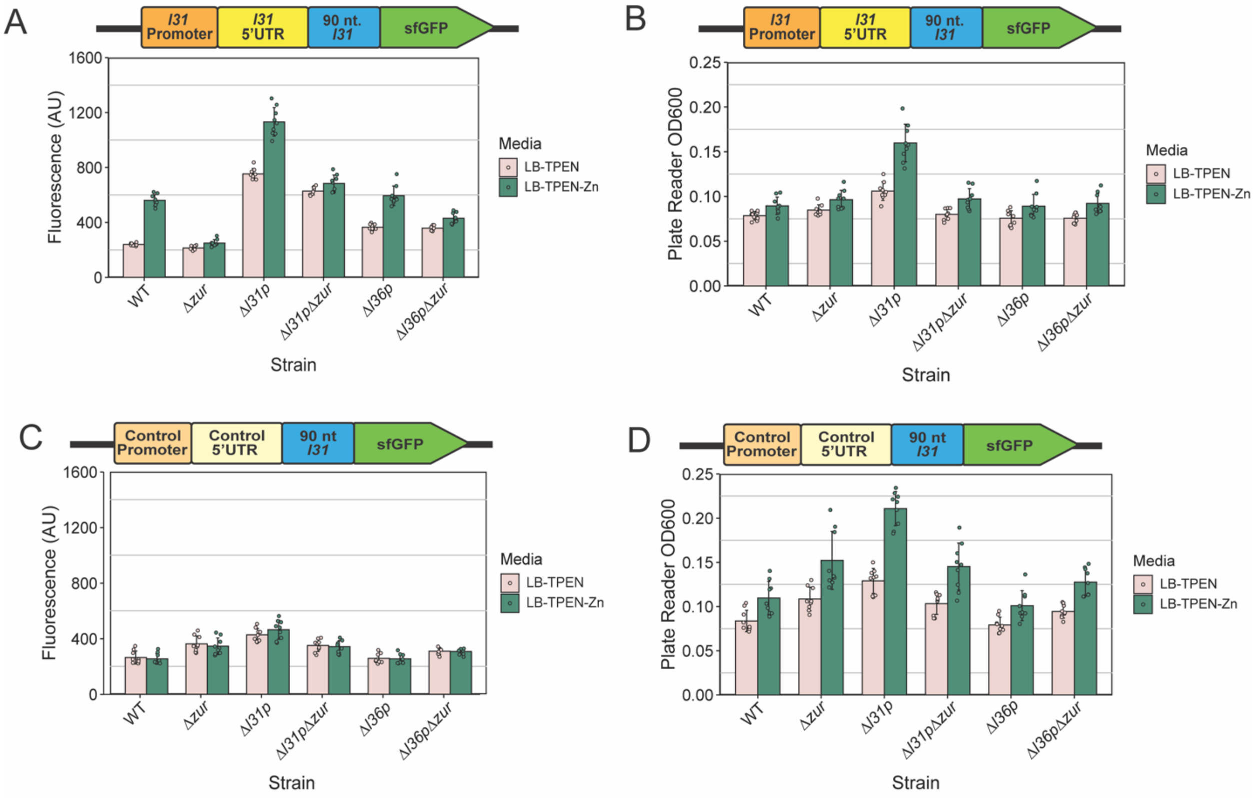

Figure S4. Growth of strains in zinc-deficient and zinc-sufficient conditions. A.) Fluorescence (AU) and B.) Plate reader OD_{600} of various strains with sfGFP plasmid, grown in LB + 100 μM of TPEN for 2 hours, then with or without the addition of 100 μM $ZnSO_4$ for an additional 2 hours. C.) Fluorescence (AU) and D.) Plate reader OD_{600} of various strains with control sfGFP-plasmid, grown in LB + 100 μM of TPEN for 2 hours, then with or without the addition of 100 μM $ZnSO_4$ for an additional 2 hours. In each graph, the points indicate averages of three biological replicates (independent experiments), each performed with three technical replicates (cultures per experiment) for a total of nine data points (n=9.) The error bars represent standard deviation of the mean.

L31 (pRAR017-L31-sfGFP)

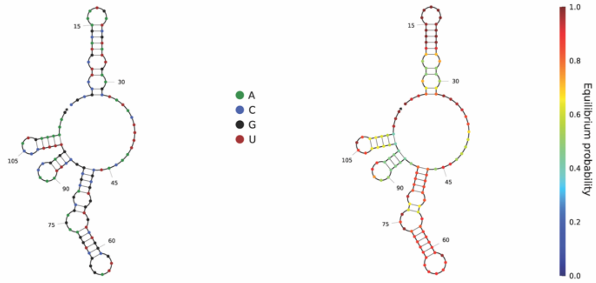

Free energy of secondary structure: -28.80 kcal/mol

Free energy of secondary structure: -28.80 kcal/mol

Scrambled Control (pRAR074-control-sfGFP)

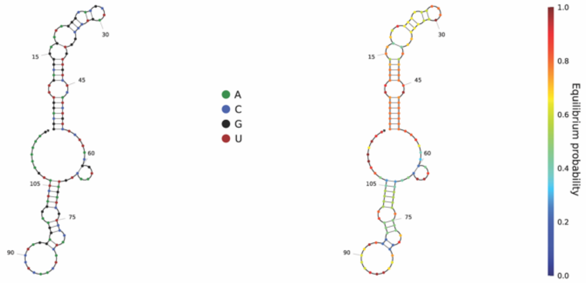

Free energy of secondary structure: -26.10 kcal/mol

Free energy of secondary structure: -26.10 kcal/mol

Δ1-31 (pRAR039)

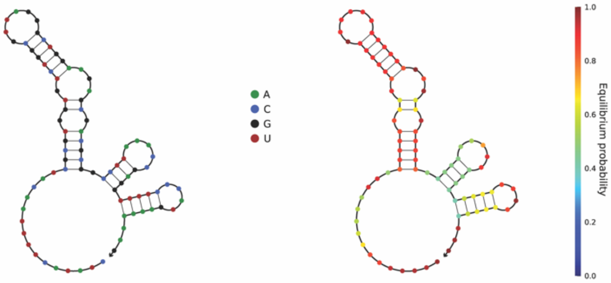

Free energy of secondary structure: -19.50 kcal/mol

Free energy of secondary structure: -19.50 kcal/mol

Δ35-46 (pRAR040)

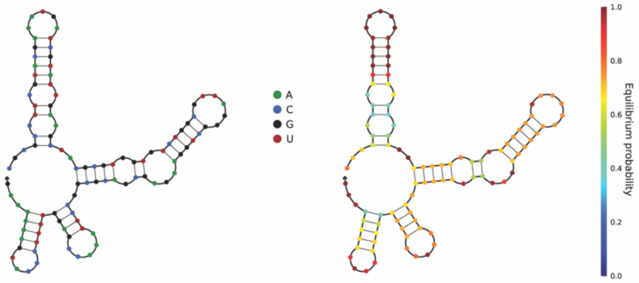

Free energy of secondary structure: -29.20 kcal/mol

Free energy of secondary structure: -29.20 kcal/mol

Δ47-54, Δ76-86 (pRAR041)

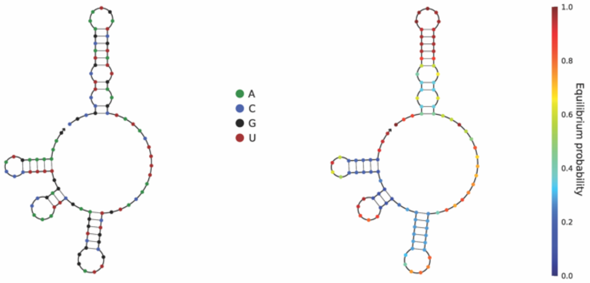

Free energy of secondary structure: -19.70 kcal/mol

Free energy of secondary structure: -19.70 kcal/mol

ΔG52 ΔG79 (pRAR105)

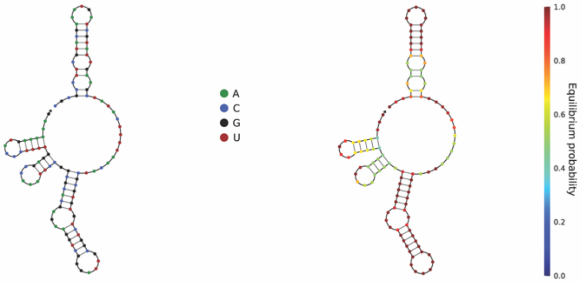

Free energy of secondary structure: -31.40 kcal/mol

Free energy of secondary structure: -31.40 kcal/mol

G79U (pRAR106)

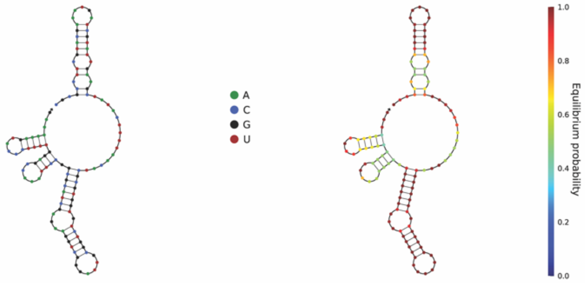

Free energy of secondary structure: -32.30 kcal/mol

Free energy of secondary structure: -32.30 kcal/mol

ΔA74 ΔA76 (pRAR104)

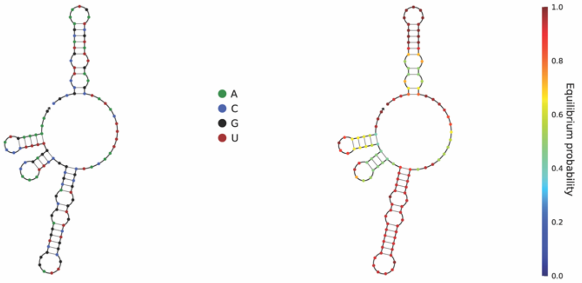

Free energy of secondary structure: -31.50 kcal/mol

Free energy of secondary structure: -31.50 kcal/mol

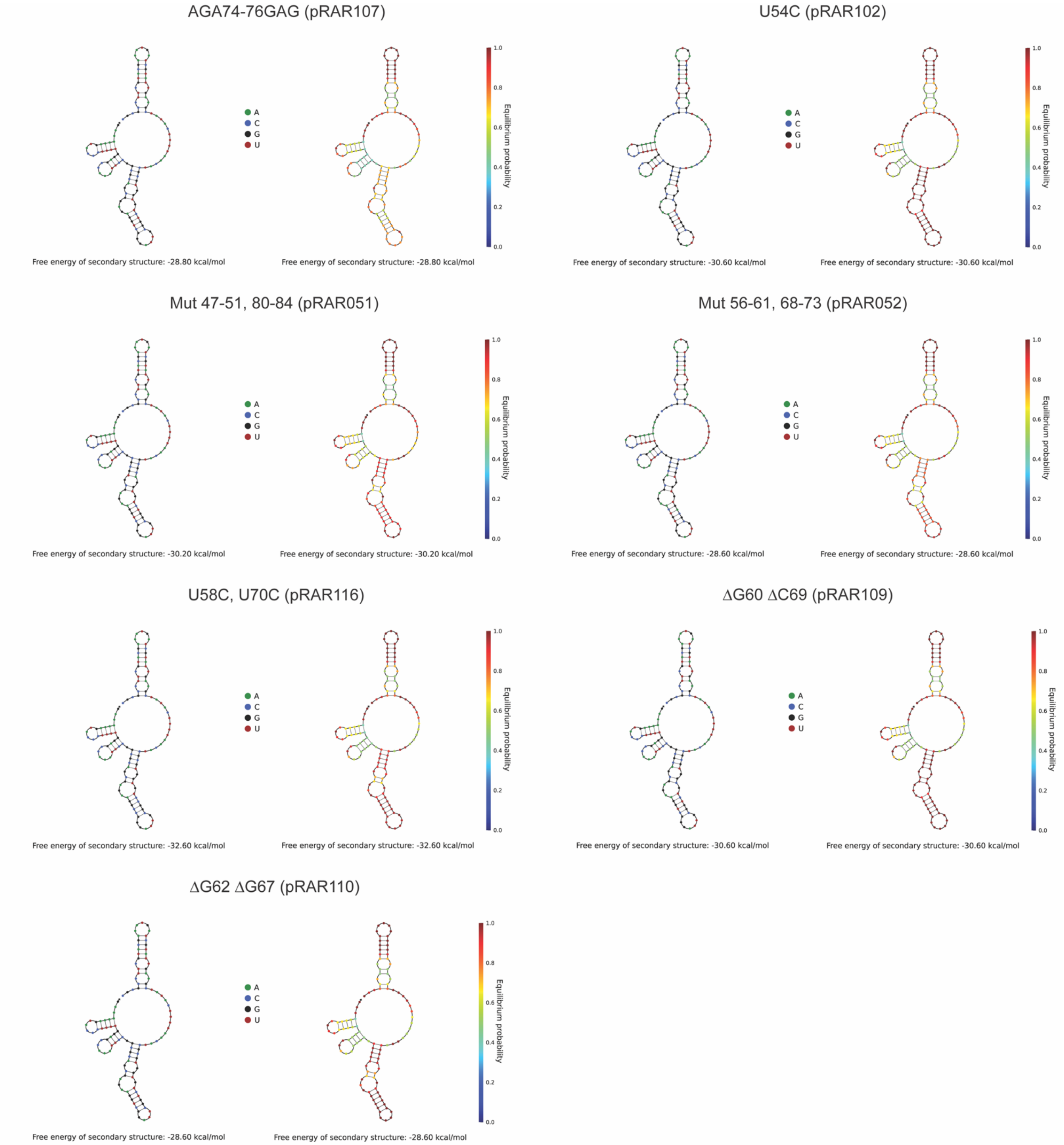

Figure S5. Predicted secondary structures of the *l31* 5'UTR and its mutants. Structures were generated using the webserver NUPACK, showing both sequence identity (left for each) and secondary structure probability (right for each). Parameters were set at temperature = 37 °C, number of strand species = 1, and maximum complex size = 1 strand.

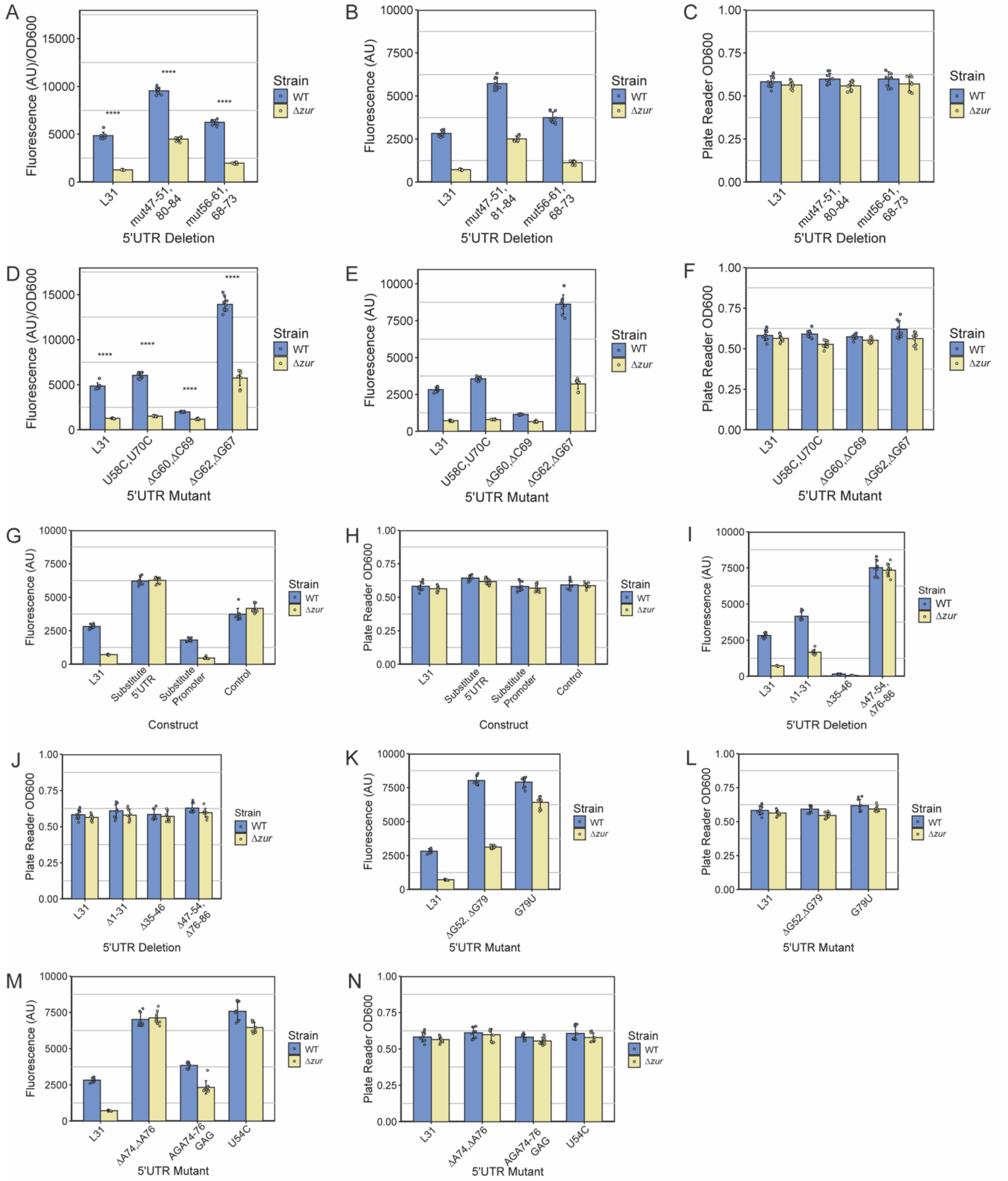

Figure S6. Some mutations to the *l31* 5’UTR affect regulation of L31-sfGFP by *zur*. Fluorescence/OD_{600} (A and D,) sfGFP Fluorescence (B, E, G, I, K, M), and Plate Reader OD_{600} (C, F, H, J, L, N) of cells grown in LB with *l31* 5’UTR mutations in L31-sfGFP plasmids. The bars indicate averages of three biological replicates (independent experiments), each performed with three technical replicates (cultures per experiment) for a total of nine data points (n=9.) The error bars represent standard deviation of the

mean. Significance was calculated with a 2-tailed student's t-test between the fluorescence/OD_{600} values for no added zinc vs added zinc for each strain. p-value < 0.05 = *, p-value < 0.01 = **, p-value < 0.001 = ***, p-value < 0.0001 = ****.

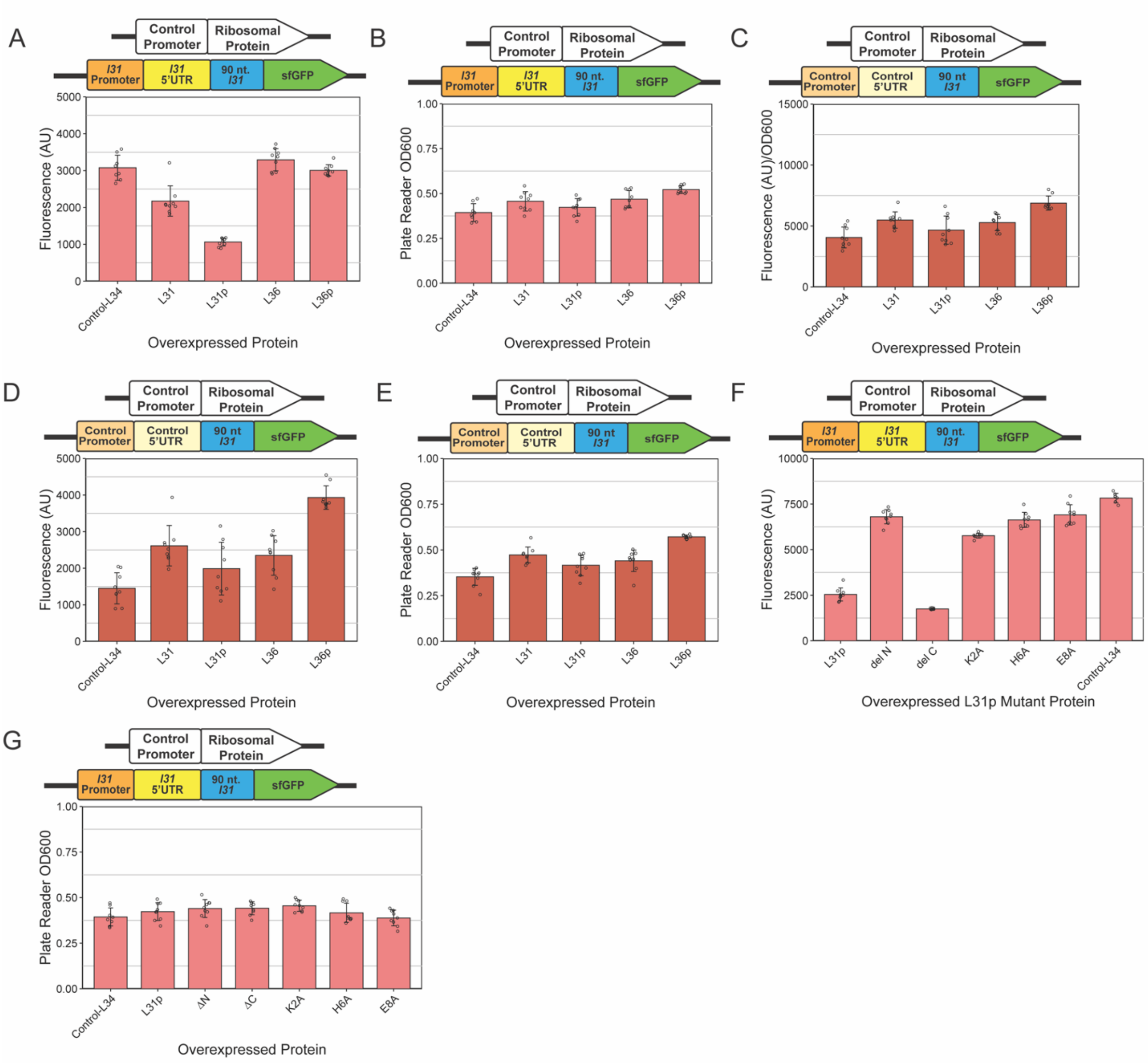

Figure S7. ***In vivo*** **sfGFP gene assays including plasmids that constitutively overexpress ribosomal proteins.** Cells were grown in LB for 6 hours. A.) Fluorescence (AU) and B.) OD_{600} measured on a plate reader for WT *E. coil* overexpressing various ribosomal proteins alongside the L31-sfGFP reporter plasmid. C.) Fluorescence/OD600 with the control-sfGFP reporter plasmid D-E.) Same as A-B.) but replacing the L31-sfGFP reporter plasmid with the control reporter plasmid. F.) Fluorescence (AU) and G.) OD_{600} measured on a plate reader, WT cells overexpressing L31p protein mutants. The bars indicate averages of three biological replicates (independent experiments), each performed with three technical replicates (cultures per experiment) for a total of nine data points (n=9). The error bars represent standard deviation of the mean.

Table S1. Plasmids used in this study.

Plasmid	Simple Name	Function
pRAR017-L31-sfGFP	L31	Reporter L31-sfGFP plasmid
pRAR039	Δ1-31	Reporter L31-sfGFP plasmid with nucleotides (nt) 1-31 of *l31* 5'UTR deleted
pRAR040	Δ35-46	Reporter L31-sfGFP plasmid with nt 35-46 of *l31* 5'UTR deleted
pRAR041	Δ47-54, 76-86	Reporter L31-sfGFP plasmid with nt 47-54 & 76-86 of *l31* 5'UTR deleted
pRAR051	Mut 47-51, 80-84	Reporter L31-sfGFP plasmid with nt 47-51 & 80-84 of l31 5'UTR mutated
pRAR052	Mut 56-61, 68-73	Reporter L31-sfGFP plasmid with nt 56-61 & 68-73 of *l31* 5'UTR mutated
pRAR064	Substitute 5'UTR	Reporter L31-sfGFP plasmid with 5'UTR replaced with scrambled control
pRAR074-control-sfGFP	Control	Reporter L31-sfGFP plasmid with promoter replaced with constitutive J23108 and 5'UTR replaced with scrambled control
pRAR089-L36p		*In vivo* expression of L36p with constitutive promoter J23108
pRAR090-L36		*In vivo* expression of L36 with constitutive promoter J23108
pRAR091-L31p		*In vivo* expression of L31p with constitutive promoter J23108
pRAR092-L31		*In vivo* expression of L31 with constitutive promoter J23108
pRAR101	ΔN	*In vivo* expression of L31p with residues 2-8 deleted, constitutive promoter J23108
pRAR102	U54C	Reporter L31-sfGFP plasmid with U54C in *l31* 5'UTR
pRAR104	ΔA74 ΔA76	Reporter L31-sfGFP plasmid with A74 and A76 deleted in *l31* 5'UTR
pRAR105	ΔG52 ΔG79	Reporter L31-sfGFP plasmid with G52 and G79 deleted in *l31* 5'UTR
pRAR106	G79U	Reporter L31-sfGFP plasmid with G79U in *l31* 5'UTR
pRAR107	AGA 74-76 GAG	Reporter L31-sfGFP plasmid with AGA 74-76 GAG mutation in *l31* 5'UTR
pRAR109	ΔG60 ΔC69	Reporter L31-sfGFP plasmid with G60 and C69 deleted in *l31* 5'UTR
pRAR110	ΔG62 ΔG67	Reporter L31-sfGFP plasmid with G62 and G67 deleted in *l31* 5'UTR
pRAR111	ΔC	*In vivo* expression of L31p with residues last 8 C-terminal residues deleted, constitutive promoter J23108
pRAR112	K2A	*In vivo* expression of L31p with K2A mutation, constitutive promoter J23108
pRAR113	H6A	*In vivo* expression of L31p with H6A mutation, constitutive promoter J23108
pRAR114	E8A	*In vivo* expression of L31p with E8A mutation, constitutive promoter J23108
pRAR116	U58C, U70C	Reporter L31-sfGFP plasmid with U58C and U70C in *l31* 5'UTR
pRAR119	L34-control	*In vivo* expression of L34 with constitutive promoter J23108
pRAR120	Substitute promoter	Reporter L31-sfGFP plasmid with promoter replaced J23108

Table S2. Primers used in RT-qPCR.

Primer	Sequence
sfGFP reverse transcription	TTATTTGTAGAGCTCATCCATG
16S rRNA reverse transcription	TAAGGAGGTGATCCAACCG
sfGFP qPCR forward	CACTGGAGTTGTCCCAATTCT
sfGFP qPCR reverse	TCCGTTTGTAGCATCACCTTC
16S rRNA qPCR forward	GTCAGCTCGTGTTGTGAAATG
16S rRNA qPCR reverse	CCCACCTTCCTCCAGTTTATC

Table S3. Overview of results of reporter gene assay figures and results in this study. This table summarizes whether results suggest that different components of the proposed mechanism are needed for *zur*'s and zinc's regulation of L31-reporter fluorescence.

Component	Needed for *zur* regulation	Needed for zinc regulation?
Genomic *zur*	--	Yes, Figure 2A
Genomic *l31p*	Yes, Figure 4A	Yes, Figure 5A
Genomic *l36p*	Likely no, Figure 4A	Likely no, Figure 5A
L31 promoter on L31-sfGFP	Likely no, Figure 6B	NA
L31 5'UTR on L31-sfGFP	Yes, Figure 6B	NA

Table S4. ICP-MS measurements of Zn in LB media used in zinc-depletion experiments.

ICP-MS LB sample	Zn64 in 7.5x diluted sample (ppb)	Zn66 in 7.5x diluted sample (ppb)	Calculated Zn (64 & 66 averaged) in undiluted sample (μM)
**1**	105.052915	103.895335	**12.15**
**2**	104.282272	102.960177	**12.01**
**3**	104.889405	105.219983	**12.16**
**Average**	105.052915	103.895335	**12.11**

Table S5. Quantitative cycle threshold values (Cq) from RT-qPCR measurements on WT and *Δzur* cells with sfGFP-plasmids.

Strain	Plasmid	Probe	Replicate	Cq
WT	L31-sfGFP	sfGFP	1	18.0656661
WT	L31-sfGFP	sfGFP	1	18.1188288
WT	L31-sfGFP	sfGFP	1	18.0517428
WT	L31-sfGFP	sfGFP	2	17.2920031
WT	L31-sfGFP	sfGFP	2	17.2825117
WT	L31-sfGFP	sfGFP	2	17.1478689
Δzur	L31-sfGFP	sfGFP	1	18.6933492
Δzur	L31-sfGFP	sfGFP	1	19.0031478

Δzur	L31-sfGFP	sfGFP	1	18.7207368
Δzur	L31-sfGFP	sfGFP	2	18.6419382
Δzur	L31-sfGFP	sfGFP	2	18.5737867
Δzur	L31-sfGFP	sfGFP	2	18.6119242
WT	Control-sfGFP	sfGFP	1	19.5046355
WT	Control-sfGFP	sfGFP	1	19.5411392
WT	Control-sfGFP	sfGFP	1	19.5551494
WT	Control-sfGFP	sfGFP	2	20.1598618
WT	Control-sfGFP	sfGFP	2	20.1760582
WT	Control-sfGFP	sfGFP	2	20.1895993
Δzur	Control-sfGFP	sfGFP	1	20.4418771
Δzur	Control-sfGFP	sfGFP	1	20.4407893
Δzur	Control-sfGFP	sfGFP	1	20.568874
Δzur	Control-sfGFP	sfGFP	2	19.5214039
Δzur	Control-sfGFP	sfGFP	2	19.4832943
Δzur	Control-sfGFP	sfGFP	2	19.5156013
WT	L31-sfGFP	16S	1	21.1197908
WT	L31-sfGFP	16S	1	21.1059921
WT	L31-sfGFP	16S	1	21.0241491
WT	L31-sfGFP	16S	2	19.1865947
WT	L31-sfGFP	16S	2	19.0946548
WT	L31-sfGFP	16S	2	18.9891716
Δzur	L31-sfGFP	16S	1	20.5732134
Δzur	L31-sfGFP	16S	1	20.3426874
Δzur	L31-sfGFP	16S	1	20.3182623
Δzur	L31-sfGFP	16S	2	19.8445053
Δzur	L31-sfGFP	16S	2	19.72594
Δzur	L31-sfGFP	16S	2	19.6529951
WT	Control-sfGFP	16S	1	20.5370341
WT	Control-sfGFP	16S	1	20.6472348
WT	Control-sfGFP	16S	1	20.5938078
WT	Control-sfGFP	16S	2	21.095067
WT	Control-sfGFP	16S	2	21.0818818
WT	Control-sfGFP	16S	2	21.1674343
Δzur	Control-sfGFP	16S	1	21.6201876
Δzur	Control-sfGFP	16S	1	21.6453969
Δzur	Control-sfGFP	16S	1	22.6815848
Δzur	Control-sfGFP	16S	2	20.6782877

Δzur	Control-sfGFP	16S	2	20.6394376
Δzur	Control-sfGFP	16S	2	20.7826001
